# Deep Reinforcement Learning for Optimizing Restricted Access Window in IEEE 802.11ah MAC Layer

**DOI:** 10.3390/s24103031

**Published:** 2024-05-10

**Authors:** Xiaojun Jiang, Shimin Gong, Chengyi Deng, Lanhua Li, Bo Gu

**Affiliations:** 1 School of Intelligent Systems Engineering, Shenzhen Campus of Sun Yat-Sen University, Shenzhen 518107, China; jiangxj5@mail2.sysu.edu.cn (X.J.); gongshm5@mail.sysu.edu.cn (S.G.); dengchy9@mail2.sysu.edu.cn (C.D.); gubo@mail.sysu.edu.cn (B.G.); 2 Guangdong Provincial Key Laboratory of Fire Science and Intelligent Emergency Technology, Guangzhou 510006, China

**Keywords:** IEEE 802.11ah, restricted access window (RAW), deep reinforcement learning (DRL)

## Abstract

The IEEE 802.11ah standard is introduced to address the growing scale of internet of things (IoT) applications. To reduce contention and enhance energy efficiency in the system, the restricted access window (RAW) mechanism is introduced in the medium access control (MAC) layer to manage the significant number of stations accessing the network. However, to achieve optimized network performance, it is necessary to appropriately determine the RAW parameters, including the number of RAW groups, the number of slots in each RAW, and the duration of each slot. In this paper, we optimize the configuration of RAW parameters in the uplink IEEE 802.11ah-based IoT network. To improve network throughput, we analyze and establish a RAW parameters optimization problem. To effectively cope with the complex and dynamic network conditions, we propose a deep reinforcement learning (DRL) approach to determine the preferable RAW parameters to optimize network throughput. To enhance learning efficiency and stability, we employ the proximal policy optimization (PPO) algorithm. We construct network environments with periodic and random traffic in an NS-3 simulator to validate the performance of the proposed PPO-based RAW parameters optimization algorithm. The simulation results reveal that using the PPO-based DRL algorithm, optimized RAW parameters can be obtained under different network conditions, and network throughput can be improved significantly.

## 1. Introduction

With the rapid development of internet of things (IoT) applications and technologies, IoT has emerged as a pivotal enabler bridging the physical and digital realms. IoT has been widely used in industry, agriculture, healthcare, and other fields. Statistics show that IoT connected devices are expected to exceed 30 billion units by 2025, more than doubling from 13.8 billion in 2021 [1]. With the expanding scope of applications, IoT has its own set of requirements: very low power, longer-range connections, and support for a greater number of client devices per access point (AP) [2]. The fulfillment of these requirements relies on the selection of wireless communication technologies.

**Figure 1:**
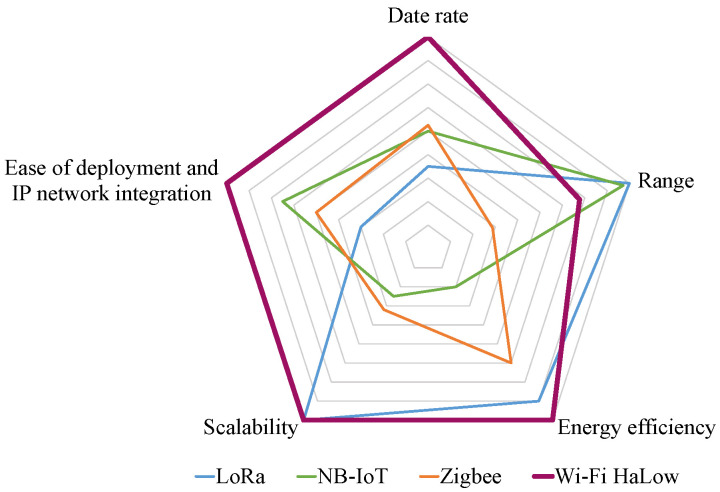
Comparison of the IEEE 802.11ah-based Wi-Fi HaLow technology with other low-power IoT technologies in terms of key aspects.

To meet the key requirements of IoT applications, the Wi-Fi Alliance has introduced Wi-Fi HaLow technology [3], which is based on the IEEE 802.11ah standard [4], operating in the unlicensed sub-1 GHz radio frequency spectrum band and utilizing narrower channels. IEEE 802.11ah is built upon the IEEE 802.11 standards with modifications for IoT applications. The physical (PHY) layer of IEEE 802.11ah is designed for long-range communication. At the medium access control (MAC) layer, novel channel access control mechanisms are introduced to facilitate access for a large number (up to 8191) of stations (STAs) and to support low power consumption. Leveraging the novel features at the PHY and MAC layers, IEEE 802.11ah offers up to 100 times longer range compared to other IoT technologies, with a data rate ranging from approximately 150 kbps to a maximum of around 86.7 Mbps [4]. As shown in Figure 1, IEEE 802.11ah-based Wi-Fi HaLow technology provides a well-balanced combination of data rate, coverage range, and energy efficiency, outperforming low-power IoT technologies such as LoRa, NB-IoT, and Zigbee [3]. Wi-Fi HaLow also features easier deployment and integration into IP networks compared to other technologies, with scalability similar to LoRa. Therefore, Wi-Fi HaLow is well-suited to meet the key requirements of IoT applications.

**Figure 2:**
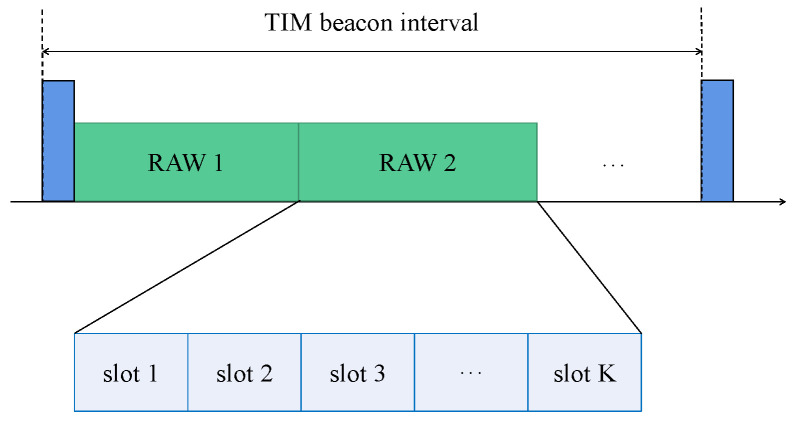
A simple demonstration of RAW.

In the MAC layer, the restricted access window (RAW) mechanism is introduced to manage the significant number of STAs accessing the network [4]. The idea of RAW is to divide the channel time into one or more access windows, where only some of the STAs can access the channel in the designated access windows, while the others are restricted from random access. As shown in Figure 2, for STAs with certain traffic patterns, the AP divides them into one or more RAW groups during a traffic indication map (TIM) beacon interval. On the arrival of each RAW, the STAs assigned to the current RAW have the right to access the channel for data transmission, while the other STAs remain dormant and cache non-urgent data until the arrival of their corresponding RAW. To further alleviate contention, each RAW is subdivided into multiple time slots with equal duration. The STAs are uniformly distributed among these slots by default. During each slot, only the STAs assigned to the current slot are permitted to contend for data transmission, ensuring that STAs restricted in different slots do not conflict with each other.

The operation of RAW mainly consists of two parts. One is the division of STAs into different RAW groups. The other is the configuration of RAW-related parameters, including the number of RAW groups, the number of slots in each RAW, the duration of each RAW, and the number of STAs in each RAW group. Different RAW parameters can change the users’ transmission strategies and thus influence the network performance, such as network throughput, latency, and energy efficiency [5,6]. However, details about RAW parameters setting and RAW grouping are not specified in the IEEE 802.11ah standard. This allows researchers the flexibility to customize the RAW configuration to meet the specific requirements of different application scenarios. Moreover, the performance of RAW can be validated in an NS-3 simulator. In [5], the authors constructed simulation environments for IEEE 802.11ah sensor networks in an NS-3 simulator that closely resembled real-world network conditions. Through simulations, detailed analyses of the impacts of RAW parameters (i.e., number of RAW groups, RAW group duration, and station division) on network throughput, transmission delay, and energy consumption have been conducted in the literature. The experiments in [6] also revealed that network performance largely depends on these RAW parameters settings.

Based on this observation, some studies have focused on finding the optimal RAW parameters to improve network performance. Researchers have conducted complicated mathematical models and have proposed heuristic methods to determine the optimal RAW parameters or grouping scheme [7,8,9]. However, most of the analytical models fail to consider the complexities and dynamic changes of network conditions, leading to discrepancies between the results derived by analytical models and those obtained from an NS-3 simulator. Moreover, heuristic methods for optimizing RAW parameters are often constrained by specific assumptions, such as fixed network topologies and known traffic patterns. The applicability of these methods in various scenarios requires further validation. Therefore, this paper aims to propose a flexible model-free learning method for finding the optimal RAW parameters, which is scalable, robust, lightweight, and capable of generalizing across different scenarios.

Due to high efficiency and strong generalization capabilities, artificial intelligence (AI) methods have found broad applications in wireless networks in recent years. There is a growing number of studies employing AI methods to solve RAW parameters optimization and grouping problems. Researchers in [10] used neural networks to decide the optimal number of RAW groups and the number of slots in each RAW for given network conditions. Moreover, machine learning (ML) methods such as K-means have been used to solve grouping problems [11]. It is noteworthy that deep reinforcement learning (DRL) integrates deep learning (DL) and reinforcement learning (RL) by using deep neural networks (DNNs) to approximate value functions or optimal policies, thereby enabling the handling of high-dimensional and complex state and action spaces. DRL’s strong performance in dealing with complex and dynamic environments endows it with powerful generalization capability, making it widely applied in wireless networks for solving parameterized optimization problems such as resource allocation and scheduling [12,13]. Therefore, it is feasible to employ the DRL approach to solve RAW parameters and network performance optimization problems.

In this paper, we propose a DRL method referring to the proximal policy optimization (PPO) algorithm to optimize the configuration of RAW parameters including the number of RAW groups, the number of slots in each RAW, and the duration of each slot, in the uplink IEEE 802.11ah-based IoT network. To improve the AP’s data collection, we aim to enhance the throughput for the overall network. Note that the proposed model-free PPO-based DRL algorithm is flexible and capable of generalizing across different scenarios. It can be easily extended to other RAW parameter optimization problems that aim to enhance other performance metrics, such as latency and energy efficiency. Specifically, we propose an efficient DRL algorithm to optimize RAW parameters to enhance network throughput. We construct different network environments using an NS-3 simulator and evaluate the learning performance of the proposed PPO-based algorithm in different scenarios. To the best of our knowledge, there are limited prior studies on RAW mechanism optimization using DRL-based approaches. Our study can serve as a reference for applying DRL to the RAW mechanism and further extensions for optimizing other mechanisms of IEEE 802.11ah. We summarize our contributions as follows:

- Performance modeling for RAW parameters optimization: The impact of RAW parameters on network throughput in the uplink IEEE 802.11ah-based IoT network is studied. To optimize network throughput, a performance analytical model is established, and a RAW parameter optimization problem is formulated.
- Guiding PPO-based DRL with NS-3 simulated network environments: An efficient learning framework is proposed to interact with different network environments constructed in an NS-3 simulator. The PPO-based DRL algorithm is designed to find the preferable RAW parameters to improve network throughput. The NS-3 simulator adeptly replicates real-world network scenarios, facilitating the training of the DRL agent. Simulation results demonstrate the effectiveness of the PPO-based DRL algorithm, with significant improvements in network throughput of 80% compared to that of the default settings schemes.

The remainder of this paper is organized as follows. Prior studies on RAW-based network performance optimization and related works using AI-based methods for the RAW mechanism are presented in Section 2. In Section 3, the network model considered in this paper and the operation of RAW are elaborated, throughput modeling with respect to RAW parameters is presented, and the RAW parameters optimization problem is established. The problem is reformulated as a Markov decision process (MDP), and a PPO-based DRL algorithm for RAW parameters optimization is proposed in Section 4. In Section 5, the performance of the proposed DRL algorithm is evaluated in simulation environments built in an NS-3 simulator. Conclusions are drawn and future studies are discussed in Section 6.

## 2. Related Work

### 2.1. Analytical Modeling for RAW Mechanism

To investigate the impact of the RAW mechanism on network performance, researchers have developed several evaluation models for the RAW-based channel access process. Typically, researchers introduce characteristics of RAW into the analytical model of the distributed coordination function (DCF) of IEEE 802.11 standards [14]. Given the known number of STAs in the network, researchers analyze the transmission and collision probabilities in a single RAW slot. The analysis is then extended to one or multiple RAWs to derive formulas for calculating network performance metrics such as throughput, delay, and energy consumption [7,15,16]. These analytical models are validated by comparing the results with those obtained from an NS-3 simulator. However, they require a series of assumptions, including saturated network traffic, ideal channel conditions, and packet loss solely caused by collisions. To obtain more accurate models, researchers have further taken unsaturated traffic, heterogeneous networks, signal capture, and other network conditions into account [6,17,18].

Moreover, researchers have investigated the impact of different RAW parameters on network performance based on analytical models or simulation results. The authors in [19] pointed out that dividing more RAWs in a beacon interval period can reduce collision probability as the total number of competing STAs in each RAW group decreases. However, this also leads to increased delay, as larger RAW segmentations increase the probability of packet buffering. Similarly, the authors in [18] stated that the more slots divided in each RAW, the fewer number of STAs competing for channel access in a single slot, thereby reducing the probability of collisions. However, the time overhead increases due to the non-cross-slot-boundary setting. The authors in [5] emphasized that a longer RAW duration generally results in better throughput. However, excessively long RAW durations perform worse in terms of latency. Moreover, the duration of a RAW should be determined based on the traffic load in each RAW group. The critical impact of RAW duration on network performance was further discussed in [6].

### 2.2. Optimization in RAW Mechanism

Given the critical impact of RAW parameters on network performance, an important issue in optimizing the RAW mechanism is the optimization of RAW parameters. RAW parameters include the number of RAW groups, the number of slots in each RAW, the duration of each RAW (which can be calculated given the slot count and slot duration), and the number of STAs in each RAW group (which can be calculated given the number of RAW groups and STAs in the network). It has been validated that the optimization of RAW parameters depends on various network variables, such as number of STAs, traffic load, and traffic patterns [5]. Most of the studies are network performance optimization-oriented, in which the authors formulate RAW parameters optimization problems and obtain one or more optimal RAW parameters using various optimization methods. To jointly maximize uplink energy efficiency and delay, the authors in [7] proposed an energy-delay-aware-window control algorithm based on the gradient descent method, enabling adaptive adjustment of slot count and slot duration according to the number of STAs in each RAW group. Similarly, the authors in [20] proposed a group-size-adaptive algorithm to determine the duration of each RAW. To cope with dynamic changes in the network size and heterogeneous traffic conditions in sensor networks with uplink traffic, the authors in [21] proposed TAROA, which can adaptively adjust RAW parameters according to the current (or estimated) traffic conditions and assign STAs to different RAW groups based on the estimated transmission frequency. TAROA has been further refined in [22]. Oriented towards delay-sensitive emergency alarm sensor networks and closed-loop communication scenarios, the authors in [23] proposed a RAW parameters selecting algorithm to minimize channel time-sharing consumption. Additionally, in [8], the authors formulated the optimal RAW scheduling problem as an integer nonlinear programming problem with the objective of minimizing channel time at key STAs and designed a heuristic algorithm to find the optimal RAW configurations.

Moreover, some studies have focused on RAW grouping, which allocates STAs to different RAW groups based on the various characteristics of the STAs. According to the priority level of the STAs, the authors in [24] proposed a QoS-aware priority grouping and scheduling algorithm. Considering the traffic characteristics (e.g., traffic demand, multi-rate) of STAs in heterogeneous networks, the authors in [25] proposed MoROA, which employs mathematical methods to solve the grouping problem and to determine the optimal RAW configurations. To achieve fairness in inter-group throughput and channel utilization the authors in [9,26] proposed heuristic grouping algorithms. Furthermore, in [27,28], the authors introduced grouping strategies based on greedy algorithms and on genetic algorithms, respectively.

### 2.3. AI-Based Methods for RAW Mechanism

It is noteworthy that in recent years there has been a growing number of studies employing AI methods to solve RAW parameter optimization and grouping problems. The authors in [29] proposed a surrogate model for RAW performance in realistic IoT scenarios by integrating ML methods such as support vector machine and artificial neural networks (ANNs). This model accurately predicts network performance for given RAW configurations in heterogeneous networks. The predicted values can serve as inputs for real-time RAW parameters optimization algorithms, thereby enhancing algorithm accuracy. In [10], the authors used ANNs to find the optimal number of RAW groups given the network size, data rate, and RAW duration. Using ML methods such as K-means, the authors implemented traffic classification and grouping schemes that can dynamically adapt to various network conditions (e.g., received signal strength, multiple rates, traffic load, and traffic arrival interval) [11,30,31,32]. In a recent study [33], the authors employed a recurrent neural network based on gated recurrent units to estimate the optimal number of RAW slots, enhancing the performance in dense IEEE 802.11ah IoT network. To the best of our knowledge, there are limited prior studies using DRL methods for RAW mechanism optimization.

## 3. RAW Mechanism in Wireless IoT Networks

**Figure 3:**
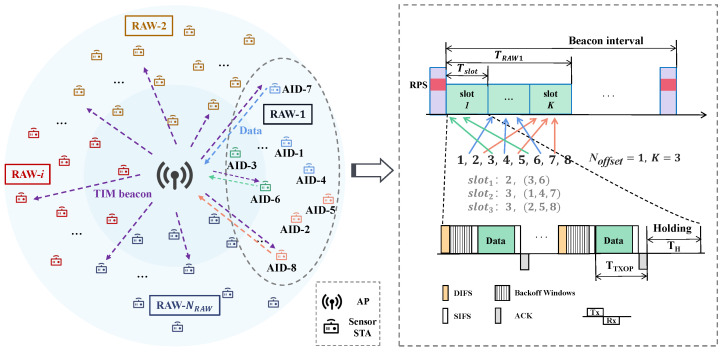
The IEEE 802.11ah-based IoT network model with RAW operations.

In this paper, we consider uplink data transmissions in a wireless IoT network employing the RAW mechanism. As shown in Figure 3, the network consists of one center-located AP and *N* randomly distributed STAs within a coverage range of several hundred meters. The STAs transmit sensory data to the AP using a specific channel access control protocol. The network traffic includes periodically generated data, as well as randomly generated data following a certain probability distribution. Since the IEEE 802.11ah standard is an ideal choice for low-power IoT networks, we employ the IEEE 802.11ah-based RAW mechanism for multiple STAs access. We further describe the RAW mechanism operating in the IoT network.

### 3.1. Operation of the RAW Mechanism

In Section 1, we briefly introduced the idea of RAW. In this section, we elaborate on the RAW parameter set involved in RAW configuration and the channel access process based on RAW in a beacon interval. We aim to explain how key RAW parameters influence network performance at the mechanism principle level.

#### 3.1.1. Structure of the RAW Parameter Set

The IEEE 802.11ah standard defines an information element field in the beacon frame for group-based restricted channel access, known as the RAW parameter set (RPS) [4]. In general, the operation of RAW is mainly implemented through the definition of the RPS in a TIM beacon, the slot allocation scheme, cross slot restrictions, and other necessary mechanisms. In IEEE 802.11ah networks, once the STAs join the network and are assigned their association identifier (AID) they listen for TIM beacon frames that carry the RPS elements, which are periodically broadcast by the AP. Consequently, the STAs in the network can know exactly the status of RAW and and their membership in a RAW group, enabling them to perform channel access and data transmission accordingly.

Specifically, RPS primarily consists of one or more RAW assignment subfields. Each RAW assignment subfield contains necessary RAW control subfields, RAW slot definition subfields, and RAW grouping subfields, for performing restricted channel access to one or multiple STAs in a RAW. According to specific requirements, elements such as RAW start time, channel indication, and periodic operation parameters subfields are conditionally present. The RAW slot definition subfield further defines the slot duration, slot count, and access restrictions between slots. As beacon frames are broadcast by the AP, STAs can learn from the related subfields of the RPS element which RAW group they belong to, as well as the number of RAW groups in a beacon interval, the number of slots in each RAW, and the duration of a single slot in each RAW. The specific rules for calculation are described as follows.

*(1) Slot duration and slot count:* The formula for calculating the duration of a single slot in a RAW is as follows [4]:

(1) $$T_{slot}=500us+C\times 120us.$$

Let the length of the slot duration count field be *y*. According to the IEEE 802.11ah standard, when y=11 bits, $C=2^{11}-1=2047$, the maximum duration of a slot is $T_{slot}^{max}=246.14$ ms and the maximum number of slots in a RAW is $K_{max}=2^{14-y}=7$. When y=8 bits, $C=2^{8}-1=255$, $T_{slot}^{max}=31.1$ ms, $K_{max}=2^{14-y}=63$. The selection of *y* depends on the number of STAs in each RAW. Apparently, the duration of a RAW can be calculated as $T_{RAW}=K\cdot T_{slot}$.

*(2) Slot assignment:* A mapping method for allocating STAs into the corresponding slots in a RAW is defined in the IEEE 802.11ah standard [4]. It is implemented by defining a mapping function,

(2) $$i=f\left(x\right)=\left(x+N_{offset}\right)\;\mathrm{mod}\;K,$$

where *x* is the AID of the STA in a RAW group, $N_{offset}$ is the allocation offset, which means that the first STA in the group will be allocated to the $N_{offset}-th$ time slot, and *K* is the number of slots in a RAW.

We provide an illustration of slot allocation in a RAW as shown in Figure 3. We assume that a RAW group division scheme configured in the RPS divides a beacon interval into $N_{RAW}$ RAW groups, with potentially different numbers of STAs, slots, and slot durations in each group. Based on the RPS settings, STAs with AIDs 1 to 8 are assigned to RAW-1 in order, with the first STA in RAW-2 being AID-9, and so on. In the RAW groups, STAs are sequentially assigned to different slots according to the mapping function. We assume the mapping offset $N_{offset}=1$ and the number of slots K=1. Consequently, in RAW-1, two STAs (with AID-3 and AID-6) are assigned to Slot-1, three STAs (with AID-1, AID-4, and AID-7) are assigned to Slot-2, and four STAs (with AID-2, AID-5, and AID-8) are assigned to Slot-3. The mapping function ensures a uniform distribution of STAs across slots.

*(3) Cross slot boundary:* The IEEE 802.11ah standard defines restrictions on channel access across slot boundaries. STAs can access the channel either in a cross-slot-boundary way or in a non-cross-slot-boundary (NCSB) way [4]. To alleviate the hidden nodes problem and facilitate performance analysis, it is generally advisable to employs the non-cross-slot-boundary mechanism [16]. Therefore, the holding time is defined to be $T_{H}\geq T_{TXOP}$, where $T_{TXOP}$ is the time required for one successful data transmission, and its expectation can be obtained through statistical analysis. With this constraint, it can be ensured that the last data transmission in the current slot has been completed by the end of slot. If the time remaining in the current slot is not sufficient for one data transmission, the STAs cache their data and wait for the arrival of the next slot to which they belong.

#### 3.1.2. RAW-Based Channel Access and Data Transmission

The channel access and data transmission process of STAs in an IEEE 802.11ah network with a RAW mechanism can be summarized as follows and is shown in Figure 3.

1. The STAs listen to the beacon frames broadcast by the AP, request association and authentication, and receive their AID. The AP periodically broadcasts beacon frames carrying the RPS element and informs the STAs of information including their RAW group, the slot count in a RAW, and the slot duration. The STAs are then assigned to different slots based on the mapping function (2).
2. The STAs contend for channel access following the enhanced distributed channel access (EDCA) mechanism when their slot arrives: the STAs perform carrier listening for a distributed inter-frame spacing (DIFS) time before initiating channel access. Once the channel is sensed to be idle, the STAs start decreasing their backoff counter, and they initiate channel access when their backoff counter reaches zero. If $STA_{a}$’s backoff counter decreases to zero before $STA_{b}$’s, $STA_{a}$ initiates channel access, while $STA_{b}$ suspends its backoff counter until the channel is sensed to be idle again.
3. If the backoff counters of two or more STAs in the network decrease to zero simultaneously, these STAs attempt to access the channel at the same time, which may result in collisions. Upon encountering a collision, the STAs increase and reset their backoff counter until they reach the maximum retry limit, at which point packet loss occurs.
4. STAs that successfully access the channel will transmit their data after waiting for a short inter-frame spacing (SIFS) time. A received acknowledgment (ACK) frame from the AP indicates the completion of data transmission. The time taken for one data transmission is denoted as $T_{TXOP}$.

The operation of the RAW mechanism elaborated above can provide a preliminary explanation at the mechanism level for the significant impact of RAW parameters on network performance: Firstly, when the number of STAs in the network is given, the number of RAW groups and the number of slots in each RAW jointly determine the number of STAs contending for channel access in a slot. Constrained by the DCF mechanism, a large number of STAs contending for channel access in a slot will intensify collisions among STAs, thereby affecting system throughput. Moreover, the duration of a slot determines the maximum number of data transmissions that can occur in each slot. When the network size increases, inadequate slot duration will limit the amount of data that STAs can transmit per slot, consequently reducing overall throughput. Due to the limitation of the NCSB mechanism, an excessive number of slots can result in frequent slot boundary switches, which in turn increases the holding time overheard and the data buffering. In general, RAW parameters, including the number of RAW groups, the number of slots per RAW, and the duration of each slot in a RAW, significantly influence network throughput. In the next subsection, we will analyze the impact of RAW parameters on network throughput at the mathematical analysis level.

### 3.2. Performance Modeling for RAW Parameters Optimization

We assume that the number of RAW groups is denoted as $N_{RAW}$, the number of slots in each RAW group is represented by $k_{i}$, and the duration of a slot in each RAW group is denoted as $t_{i}$, where $i\in \left[1,N_{RAW}\right]$. Thus, the set of the number of STAs in each RAW group, the set of the number of slots in each RAW, and the set of slot durations for RAW groups are represented as $N_{STA}=\left\{n_{1},\ldots ,n_{i},\ldots ,n_{N_{RAW}}\right\}$, $K_{RAW}=\left\{k_{1},\ldots ,k_{i},\ldots ,k_{N_{RAW}}\right\}$, and $T_{RAW}=\left\{t_{1},\ldots ,t_{i},\ldots ,t_{N_{RAW}}\right\}$, respectively.

The correlation between RAW parameters and network throughput can be derived based on the analytical model proposed in [14]. Given that the STAs are uniformly distributed among slots in a RAW, the number of STAs in each slot can be approximated as $x_{i}=\frac{n_{i}}{k_{i}}$, and the intensity of contention in each slot is considered to be the same. Consequently, for the STAs in each slot of the *i*-th RAW, the probability of STAs suspending their backoff counter is defined as $p_{f,i}\left(\tau_{i},x_{i},t_{i}\right)$, indicating that the suspending probability is related to the transmission probability $\tau_{i}$, the number of STAs in each slot $x_{i}$, and the slot duration $t_{i}$. The collision probability is denoted as $p_{c,i}$.

The backoff process of an STA’s backoff counter can be analyzed using a two-dimensional Markov chain [14]. Each state during the backoff process can be represented as a probability, and the steady-state probability of each state can be further determined. According to the normalization formula, a closed-form expression for the steady-state probability of the backoff counter decreasing to zero can be obtained as $b_{i,0}\left(p_{f},p_{c},CW_{min},m\right)$, indicating that the steady-state probability at state-0 is dependent on the suspending probability $p_{f,i}$, the collision probability $p_{c}$, the given minimum size of the contention window $CW_{min}$, and the retry limit *m*. Subsequently, the transmission probability can be computed as

(3) $$\tau_{i}=\frac{1-{\left(p_{c,i}\right)}^{m+1}}{1-p_{c,i}}b_{i,0}.$$

The collision probability is given by $p_{c,i}=1-{\left(1-\tau_{i}\right)}^{x_{i}-1}$, and the probability that at least one STA transmits data in a slot is denoted as $P_{tr,i}=1-{\left(1-\tau_{i}\right)}^{x_{i}}$. Furthermore, the successful transmission probability can be represented as

(4) $$P_{suc,i}=\frac{x_{i}\tau_{i}{\left(1-\tau_{i}\right)}^{x_{i}-1}}{P_{tr,i}}.$$

The normalized slot throughput can be calculated as

(5) $$u_{i}=\frac{P_{tr,i}P_{suc,i}E\left(D\right)}{\left(1-P_{tr,i}\right)\sigma +P_{suc,i}T_{suc}+p_{c,i}T_{c}},$$

where E(D) represents the average payload size of a data frame and σ is the time of a mini-slot in the contention window. The time for a successful data transmission and the time spent due to collision are denoted as $T_{suc}$ and $T_{c}$, respectively, and are calculated in [14]. The effective time for data transmissions in a slot is $t_{i}^{\prime }=t_{i}-T_{H}$. Finally, the normalized throughput of the network can be denoted by

(6) $$U=\sum_{i=1}^{N_{RAW}}\frac{u_{i}k_{i}t_{i}^{\prime }}{T_{BI}},$$

where the duration of the beacon interval $T_{BI}$ is dependent on the total duration of RAWs in one beacon interval.

According to (6), network throughput is related to successful transmission probability, which in turn depends on collision probability and transmission probability. These probabilities are influenced by the number of STAs in a slot and the slot duration. Moreover, the number of RAW groups and the number of slots in a RAW jointly determine the number of STAs in a slot. Intuitively, the increasing number of RAW groups and slot divisions reduces the number of STAs per slot, thereby decreasing the collision probability. Increasing the slot duration, on the other hand, allows more time for data transmission in a slot, thereby reducing data buffering. Therefore, increasing the number of RAW groups, dividing more slots in a RAW, and extending the slot duration can greatly enhance network throughput. However, excessive RAW divisions may cause more STAs to remain idle, leading to data buffering. Similarly, an excessively long slot duration may result in wasted time in networks with low traffic loads. There is a trade-off in adjusting the RAW parameters. Hence, by jointly optimizing the number of RAW groups $N_{RAW}$, RAW slot counts $k_{i}\in K_{RAW}$, and slot durations $t_{i}\in T_{RAW}$ with $i\in \left[1,N_{RAW}\right]$, we can formulate the network throughput maximization problem as follows:

(7) $$\begin{array}{cc} \max_{N_{RAW},\;K_{RAW},\;T_{RAW}}\;\;\; & U\\ \;\;\;\;\;\;\;\;\;\;\;s.t.\;\;\;\;\;\;\;\;\;\;\; & \left(1),\;(3),\;(4),\;(5),\;\mathrm{and}\;(6\right)\\ \; & \sum_{i}k_{i}t_{i}\leq T_{BI}. \end{array}$$

The existing studies prefer to construct complicated analytical models of RAW, and they further propose optimization methods to find the optimal RAW parameters to improve network throughput. However, solving RAW parameters optimization problems based on analytical models may lead to a high level of computational complexity or even impracticality in dynamic networks. On the one hand, these analytical models require a series of assumptions, including saturated network traffic, ideal channel conditions, and packet loss solely caused by collisions. Moreover, the analytical models do not comprehensively consider details about the RAW mechanism and channel conditions. Although some studies have refined the analytical models and taken more complex network conditions into account, this has made the analysis process more cumbersome. On the other hand, because the mathematical or heuristic methods often involve complex rules and have not been validated in different network scenarios, their generalization ability in complex and dynamic network conditions needs to be improved.

To investigate practical network states, the IEEE 802.11ah network simulation environment was developed based on a widely used network simulator called NS-3 [5]. NS-3 is used to create simulation environments that closely resemble real-world network environments. The partial mechanisms of the PHY and MAC layers including the RAW mechanism are also implemented. While analytical results serve as references for optimizing RAW parameters, the simulation environment implemented by NS-3 undoubtedly provides more accurate results and can serve as a benchmark for validating these analytical results. Additionally, with the capability of handling complex and dynamic environments, DRL-based methods are well-suited for addressing RAW parameters optimization problems and demonstrate strong generalization ability across various scenarios.

To determine the preferable RAW parameters that improve network throughput in complex network environments resembling real-world scenarios, we construct network environments using an NS-3 simulator, and employ the DRL-based method to optimize the RAW parameters for enhanced network throughput. The specific methodology will be elaborated in the following section.

## 4. DRL for RAW Parameters Optimization

**Figure 4:**
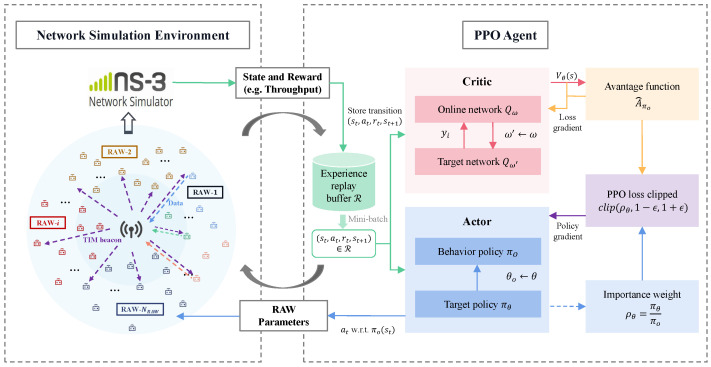
DRL framework for optimizing RAW parameters in NS-3.

In this section, we propose a learning framework for optimizing RAW parameters. As depicted in Figure 4, we set up network simulation environments in NS-3 and execute agent training in the DRL environment. The PPO algorithm [34] is employed as the specific implementation algorithm in the DRL framework for optimizing RAW parameters in NS-3, achieving enhanced learning efficiency and policy update stability. During training, the DRL agent receives network observations from the NS-3 simulation environment, serving as inputs to the DNNs. Each learned action (i.e., the RAW parameters) is then applied as the configuration parameters for the RAW mechanism in NS-3, and a new simulation is executed to obtain an updated reward (i.e., the network throughput). The DRL agent continues to receive observations from the network environment for a new training episode. Interactions between the DRL agent and the NS-3 environment continue until the DRL agent learns the preferable RAW parameters and achieves enhanced network throughput. To utilize PPO for optimizing the RAW parameters to maximize network throughput, we first reformulate problem (7) as an MDP.

### 4.1. MDP Reformulation

Given the network conditions, we aim to optimize the RAW parameters (i.e., the number of RAW groups, the number of slots in each RAW, and the slot duration in each RAW) to reduce contentions among the STAs and consequently improve the network throughput. To facilitate the problem formulation, the following assumptions are made for the IoT network:

1. The AP collects information about the network (e.g., network size *N* and traffic arrival of the STAs) through management frames. Based on received packets from the STAs, the network performance, such as throughput and packet loss ratio, can be statistically determined.
2. To alleviate hidden nodes issues and collisions, the STAs obey the NCSB mechanism when accessing the channel among slots.

In RL, the interaction between the agent and the environment is typically modeled as an MDP, which can be represented by a tuple (S,A,P,r,γ), where S represents the state space, A represents the action space, the transition probability function $P(s^{\prime }|s,a)$ represents the probability of transitioning from state *s* to state $s^{\prime }$ when action *a* is taken, the reward function r(s,a) represents the reward obtained after taking action *a* in state *s*, and γ∈[0,1] is a constant discount factor. Specifically, the definitions of state, actions, reward, and observations are given as follows:

1. State: The state at the current time step is defined as the throughput obtained from the current simulation statistics, denoted as $s_{t}=U_{t}$. During the simulation, the AP collects the number of packets received and the payload size of each packet at the current time step to calculate the network throughput at the end of the current step.
2. Action: The actions in the MDP are defined as the RAW parameters, including the number of RAW groups, the number of slots in each RAW group, and the slot duration in each RAW group. Thus, the action at step *t* is denoted as $a_{t}=\left(N_{RAW},K_{RAW},T_{RAW}\right)$.
3. Reward: According to the optimization objective, the reward is defined as the throughput obtained at each time step, represented as $r_{t}=U_{t}$.
4. Observation: The observation set is defined as the network information observable by the AP, including network size *N*, the set of traffic loads D, and the set of traffic intervals I, which can be represented as $o_{t}=\left(N,D,I\right)$.

In the following subsection, we elaborate on the PPO algorithm for RAW parameters optimization.

### 4.2. PPO for Optimizing RAW Parameters

Given a policy approximator $\pi_{\theta }\left(a|s\right)$ with parameters θ, policy-based policy gradient (PG) algorithms find the optimal θ to maximize the reward or value function [35]. For a given input state $s_{t}$, the policy network directly outputs either the action or the probability associated with the action. It then selects the appropriate action based on the probability, allowing the output action to be a continuous value. The expected value function in PG algorithms can be represented in terms of the policy parameters as

(8) $$\begin{array}{c} J\left(\theta \right)=\sum_{s}d^{\pi_{\theta }}\left(s\right)V^{\pi_{\theta }}\left(s\right)=\sum_{s}d^{\pi_{\theta }}\left(s\right)\sum_{a}\pi_{\theta }\left(s,a\right)Q^{\pi_{\theta }}\left(s,a\right), \end{array}$$

where $d^{\pi_{\theta }}$ is the stationary distribution of the Markov chain for $\pi_{\theta }$, and $Q^{\pi_{\theta }}\left(s,a\right)$ denotes the Q-value of the state–action pair (s,a) following the policy $\pi_{\theta }$. The goal of PG is to find parameters θ that maximize J(θ) by ascending the gradient of the policy. The evaluation of the policy gradient $\nabla_{\theta }J\left(\theta \right)$ can be simplified as [36]

(9) $$\begin{array}{c} \nabla_{\theta }J\left(\theta \right)=E_{\pi }\left[Q^{\pi }\left(s,a\right)\nabla_{\theta }\ln\pi_{\theta }\left(a|s\right)\right)]=E_{\pi }\left[\sum_{t=1}^{T}Q^{\pi }\left(s_{t},a_{t}\right)\nabla_{\theta }\ln\pi_{\theta }\left(a_{t}|s_{t}\right)\right)], \end{array}$$

where the expectation is taken over all possible state–action pairs following the same policy $\pi_{\theta }$. The policy gradient $\nabla_{\theta }J\left(\theta \right)$ can be evaluated by sampling historical decision-making trajectories.

For each episode, all the $\left(s,a,r,s^{\prime }\right)$ tuples acquired by the agent can be collectively represented as a state–action trajectory resulting from the agent’s interaction with the environment over the current episode, which is denoted as $\tau =\left(s_{0},a_{0},r_{1},s_{1},\ldots ,s_{T-1},a_{T-1},r_{T-1},s_{T}\right)∼\left(\pi_{\theta },P\left(s_{t+1}|s_{t},a_{t}\right)\right)$. Let $G_{t}=\sum_{k=t}^{T}r\left(s_{k},a_{k}\right)$ be the reward for a trajectory τ, and estimate the Q-value $Q^{\pi }\left(s_{t},a_{t}\right)$ in (9) by $G_{t}$. Therefore, the policy gradient in each time step can be approximated by randomly sampling $G_{t}\nabla_{\theta }\ln\pi_{\theta }\left(a_{t}|s_{t}\right))$, and the policy parameters can be updated as $\theta \leftarrow \theta +\alpha \nabla_{\theta }J\left(\theta \right)$, where α denotes the step size for the gradient update. To reduce prediction variability and improve learning efficiency, the value function $V_{\pi }\left(s\right)$ can be used as the baseline, and the advantage function $A_{\pi }\left(s,a\right)≜Q^{\pi }\left(s,a\right)-V_{\pi }\left(s\right)$ is further introduced to replace $G_{t}$.

To address high-dimensional state and action spaces while stabilizing the learning process, actor–critic (AC)-based DRL algorithms introduce a DNN with weight parameters ω to approximate the Q value. AC algorithms update both the policy network and the Q-value network. Specifically, at each learning step *t*, the actor updates the policy network by updating the policy parameters $\theta \leftarrow \theta +\alpha_{\theta }Q_{\omega }\left(s,a\right)\nabla_{\theta }\ln\pi_{\theta }\left(a|s\right))$, while the critic updates the Q network by minimizing a loss function and updates the parameters $\omega \leftarrow \omega +\alpha_{\omega }\delta_{t}\nabla_{\omega }Q_{\omega }\left(s,a\right)$ by gradient ascent, where $\delta_{t}=r_{t}+\gamma Q_{\omega }\left(s^{\prime },a^{\prime }\right)-Q_{\omega }\left(s,a\right)$ denotes the TD error. To further stabilize the training process, the deep deterministic gradient policy (DDPG) algorithm [36] utilizes two DNNs with different parameters, i.e., the online Q-network $Q_{\omega }\left(s,a\right)$ and the target Q-network $Q_{\omega^{\prime }}\left(s,a\right)$. The TD error is rewritten as $\delta_{t}=r_{t}+\gamma Q_{\omega^{\prime }}\left(s^{\prime },a^{\prime }\right)-Q_{\omega }\left(s,a\right)$.

To facilitate the agent’s utilization of past experiences and improve sample efficiency, PG can be transformed into off-policy learning through the utilization of importance sampling [37]. Sample collections can be conducted under a behavior policy $\pi_{o}\left(s,a\right)$ distinct from the target policy $\pi_{\theta }\left(a|s\right)$.

To mitigate the effects of improper step size in policy optimization on training stability, the off-policy trust region policy optimization (TRPO) algorithm imposes an additional constraint on the gradient update [37], ensuring that the old and new policies do not diverge significantly. Let $\rho_{theta}=\frac{\pi_{\theta }\left(s,a\right)}{\pi_{o}\left(s,a\right)}$ denote the probability ratio of the divergence between the old and new policies. TRPO maximizes the objective by applying conservative policy iteration without limiting the probability ratio to an appropriate range. This could lead to an excessively large policy update. Intuitively, a smaller deviation between the behavior policy and the target policy is better. Hence, the PPO algorithm [34] modifies the objective by constraining $\rho_{\theta }$ in a region [1−ϵ,1+ϵ] and penalizing changes to the policy that move $\rho_{\theta }$ away from 1. The objective in $PPO_{CLIP}$ is

(10) $$\begin{array}{cc} \max_{\theta }\;\;\; & L^{CLIP}\left(\theta \right)=\tilde{J}\left(\theta \right)=E_{\pi_{o}}\left[\min\left\{\rho_{\theta }{\hat{A}}_{\pi_{o}},clip\left(\rho_{\theta },1-\epsilon ,1+\epsilon \right){\hat{A}}_{\pi_{o}}\right\}\right]\\ s.t.\;\;\; & D_{KL}\left(\pi_{o},\pi_{\theta }\right)\leq \delta_{KL}, \end{array}$$

where $D_{KL}\left(P_{1},P_{2}\right)≜\int_{\infty }^{\infty }P_{1}\left(x\right)\log\left(P_{1}\left(x\right)/P_{2}\left(x\right)\right)\;dx$ denotes a distance measure in terms of the Kullback–Leibler (KL) divergence between two different probability distributions. The advantage function $A_{\pi_{o}}$ in the objective of problem (10) is the approximation of the actual advantage $A_{\pi_{\theta }}$ corresponding to the target policy $\pi_{\theta }$. PPO constrains the parameters search within a region by introducing the inequality constraint in problem (10), which ensures that the KL convergence between $\pi_{\theta }$ and $\pi_{o}$ is bounded by $\delta_{KL}$. The clip function returns $\rho_{\theta }\in \left[1-\epsilon ,1+\epsilon \right]$ and the hyper-parameter ϵ=0.2 by default.

During the training of the DNNs, PPO employs fixed-length (e.g., *T* time steps) trajectory segments. A truncated advantage estimation is computed to replace the advantage function in problem (10) as

(11) $${\hat{A}}_{t}^{\pi_{o}}=\delta_{t}+\gamma \delta_{t+1}+\cdots +\gamma^{T-t+1}\delta_{T-1},$$

where $\delta_{t}=r_{t}+\gamma V\left(s_{t+1}\right)-V\left(s_{t}\right)$. The loss function of PPO is

(12) $$L_{t}\left(\theta \right)=E_{t}\left[L_{t}^{CLIP}\left(\theta \right)-c_{1}L_{t}^{VF}\left(\theta \right)+c_{2}S\left[\pi_{\theta }\right]\left(s_{t}\right)\right],$$

where $L^{VF}=V_{\theta }\left(s_{t}\right)-V_{t}^{target}$ is the mean squared error loss, $c_{1},c_{2}$ are coefficients, and *S* is an entropy bonus.

The PPO algorithm for optimizing RAW parameters in networks built in NS-3 is summarized in Algorithm 1. PPO utilizes two DNNs to approximate the policy networks. In each learning episode, the PPO agent runs the old/behavior policy $\pi_{\theta_{o}}$ (i.e., RAW parameters), observes network throughput obtained from the NS-3 network simulations environment for *T* time steps, and stores *T* transition tuples $(s_{t},a_{t},r_{t},s_{t+1}),t\in T$ in the experience replay buffer. Then, it samples mini-batches of transition tuples from the replay buffer and computes advantage estimates ${\hat{A}}_{1}^{\pi_{o}},\cdots ,{\hat{A}}_{T}^{\pi_{o}}$. Subsequently, the weight parameters of the target policy network $\pi_{\theta }$ are updated by using mini-batches randomly sampled from the replay buffer through importance sampling and by optimizing the surrogate loss in (12). The weight parameters of the behavior policy network are updated by $\theta_{o}\leftarrow \theta$. Limiting the probability ratio of the two policies $\rho_{\theta }\in \left[1-\epsilon ,1+\epsilon \right]$ ensures that the probability distribution of the output actions from the two policy networks remains similar.
**Algorithm 1** PPO for RAW parameters optimizationInitialize target policy $\pi_{\theta }$ and behavior policy $\pi_{o}$Initialize online critic $Q_{\omega }$ and target critic $Q_{\omega^{\prime }}$Initialize clipping threshold ϵs**for** episode =1,…,M **do**   **while** t≠T **do**     Observe the system state $s_{t}$ from NS-3     Select an action $a_{t}$ according to behavior policy $\pi_{o}\left(s_{t}\right)$     Execute action $a_{t}=\left(N_{RAW},K_{N_{\mathrm{RAW}}},T_{N_{\mathrm{RAW}}}\right)$ in NS-3, obtain network throughput reward $r_{t}=U_{t}$, evaluate $V_{\theta }\left(s_{t}\right)$ and next state $s_{t+1}$     Store transition tuple $\left(s_{t},a_{t},r_{t},s_{t+1}\right)$ and $V_{\theta }\left(s_{t},a_{t}\right)$ in *R*     t←t+1   **end while**   Sample mini-batch of transitions $\left(s_{i},a_{i},V_{\theta }\left(s_{i},a_{i}\right),s_{i+1}\right)$ from *R*   Estimate advantage ${\hat{A}}_{\pi_{o}}$ using advantage according to (11)   Update target policy by solving problem (10)   Update behavior policy $\pi_{\theta_{o}}\leftarrow \left(1-\epsilon \right)\pi_{\theta_{o}}+\epsilon \pi_{\theta }$   Update online and target critic by minimizing the value loss in (12) using gradient descent**end for**

The uniform grouping scheme has been verified to perform better in homogeneous networks [38]. Considering the networks with periodic and random traffic in this paper, we employ the uniform grouping scheme, where STAs are evenly distributed in each RAW. Consequently, the slot duration and number of slots in each RAW group are considered to be equal. As a result, the actions of the MDP can be further simplified to the number of RAW groups, the number of slots in one RAW group, and the slot duration in one RAW group.

## 5. DRL-Guided NS-3 Simulation

In this section, we investigate the performance of the proposed PPO-based DRL algorithm for RAW parameters optimization in networks with periodic or random traffic, which are set up in the NS-3 simulator. We firstly demonstrate the learning performance of the PPO algorithm on finding preferable RAW parameters to enhance network throughput. Then, we investigate the adaptive capability of RAW parameters under dynamic network conditions such as traffic load and network size. Finally, we compare the performance of the PPO-based slot-division scheme with the equal-slot-division scheme (i.e., one STA per slot) and no-slot-division scheme (i.e., only one ’slot’ in a RAW).

### 5.1. Simulation Setup

We set up the training environment for DRL on the Linux operating system. Specifically, we set up the DRL agent in a Python environment based on the PyTorch framework, and set up the network topology in the NS-3 simulator as depicted in Figure 3. Network conditions and simulation results are input into the PPO agent as environment states. The RAW parameters are configured based on the actions learned by the PPO agent and used for subsequent simulations in the NS-3 simulator. Throughout the training process, the PPO agent interacts numerous times with the simulated network environment set up in the NS-3 simulator.

For the two different network environments established in NS-3, the two scenarios are primarily differentiated based on the network size, the traffic load of the STAs, and the traffic interval of the STAs, and are denoted as *N*, $D=\{d_{1},\ldots ,d_{N}\}$, and $I=\{i_{1},\ldots ,i_{N}\}$, respectively. These serve as the main environmental characteristics in the observations of the MDP established in Section 4.1.

The environment settings in the periodic traffic networks are summarized in Table 1. Specifically, we set small network sizes (N< 100). Each STA has the same traffic load, e.g., d=0.005 Mbps. During data transmission, the packet transmission interval of each STA follows a fixed time interval, such as i=0.001 ms. Parameters settings for the PPO agent are shown in Table 2. For analytical and simulation design purposes, we define the time step *t* as each fixed-duration simulation iteration performed in NS-3, where each episode consists of only one step. When performing simulations, we collect statistical information regarding network performance after every simulation iteration with a duration of 10 s. Note that the number of slots $K_{RAW}$ is in the range of [1,63] according to the restriction in (1). When $K_{RAW}\in \left[1,7\right]$, the maximum slot duration is 246.14 ms, and when $K_{RAW}\in \left[8,63\right]$, the maximum slot duration is 31.1 ms. This can serve as a constraint for the agent during learning.

### 5.2. Learning Performance in Periodic Traffic Networks

We first consider the RAW parameters optimization in networks with periodic traffic, where all STAs are assigned to one RAW group. The action of the MDP is $a_{t}=\left(K_{N_{RAW=1}},T_{N_{RAW=1}}\right)$. In each iteration during the training process, the PPO agent observes throughput $s_{t}$ and other information $o_{t}$ from the wireless environment and employs policy π to determine the RAW parameters setting $a_{t}$ for the next time step. The effectiveness of the RAW parameters is evaluated at the subsequent time step with the reward obtained from NS-3, and the RAW parameters for the following time step are determined accordingly. The PPO agent is trained through numerous interactions with network simulation environments built in NS-3.

#### 5.2.1. Convergence to the Preferable RAW Parameters

**Figure 5:**
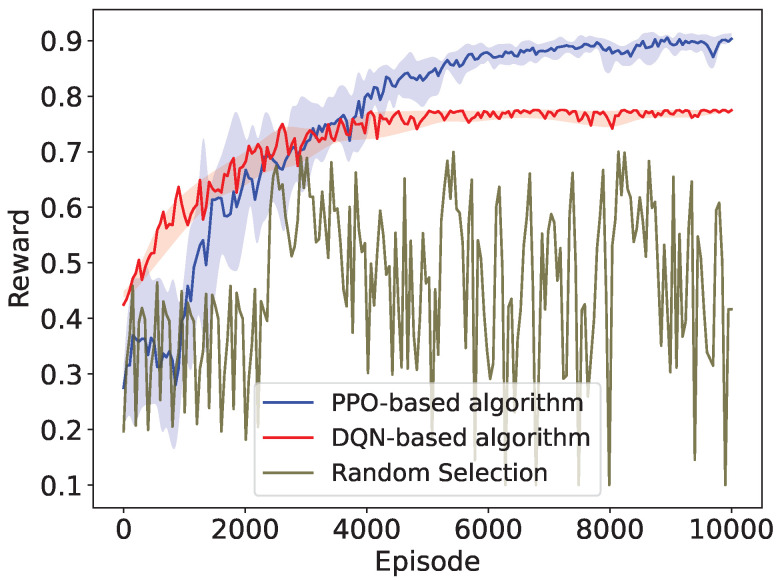
Convergence performance of the PPO-based algorithm compared with the DQN-based algorithm and the random selection scheme.

**Figure 6:**
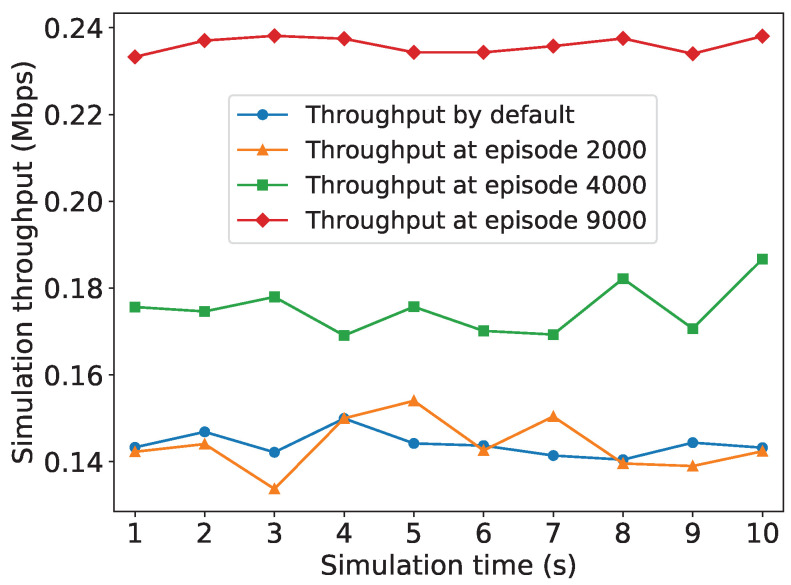
Throughput ascending in NS-3 simulation environment during training episodes.

We first validate the convergence performance of the PPO algorithm on a basic network topology. In the periodic traffic network, the traffic interval of all STAs is fixed (e.g., 0.1 ms). We train the PPO agent though numerous interactions with the network environment built in NS-3. The convergence performance of the PPO algorithm is shown in Figure 5, and Figure 6 demonstrates the convergence process of the PPO agent interacting with the network simulation environment in the NS-3 simulator. As the training iteration proceeds, the PPO agent learned better actions, leading to significantly increased normalized rewards obtained from interacting with the NS-3 simulation environment. After 10,000 training episodes, the reward stabilized at its maximum value. This indicates that the PPO agent has learned the preferable RAW parameters by the end of training and has achieved the optimized network throughput in the periodic traffic network.

We also observe the improvement of network throughput with the NS-3 simulator during the PPO agent’s training process. It can be seen in Figure 6 that the network throughput is ascending when the training process proceeds. Compared to the network throughput obtained with default settings ($K_{RAW}=1,\;TBI=100\;$ms), the network throughput with the preferable RAW parameters obtained by the PPO agent is improved by about 70%. It is evident that employing the DRL method for optimizing RAW parameters is feasible, and that the RAW parameters derived from learning lead to a significant enhancement in network throughput compared to default settings.

To further validate the effectiveness of the proposed PPO-based algorithm, we compare it with the value-based Deep Q-Network(DQN) algorithm and the random RAW parameters selection scheme. DQN is suitable for discrete action learning but struggles with high-dimensional action spaces like RAW parameters. Therefore, we apply interval sampling to reduce the action space. As shown in Figure 5, compared to random selection, both DQN and PPO can converge to stable rewards through training, outperforming the random selection scheme. This observation highlights the ability of DRL methods to optimize RAW parameters and improve network throughput. Moreover, reducing the action space accelerates the convergence of DQN, requiring 50% fewer training episodes than PPO. However, this also leads to DQN’s inferior performance, with a 20% lower stabilized reward than PPO.

**Figure 7:**
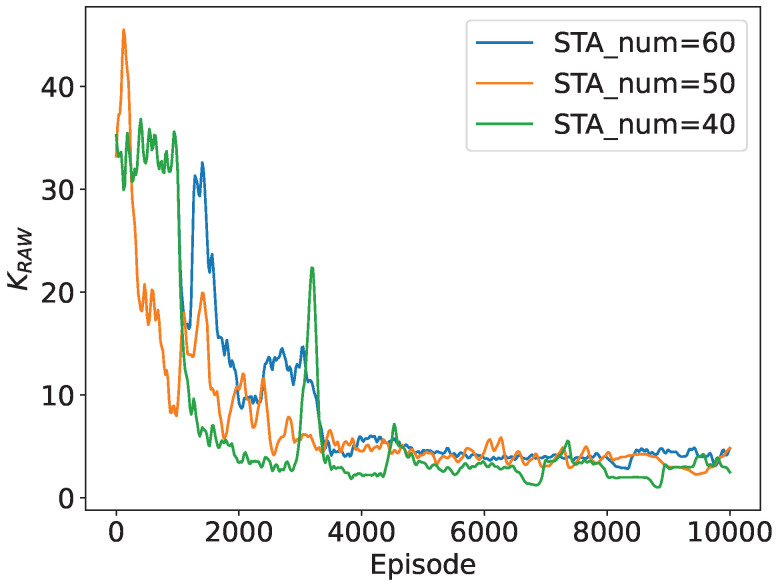
Convergence performance of $K_{RAW}$.

**Figure 8:**
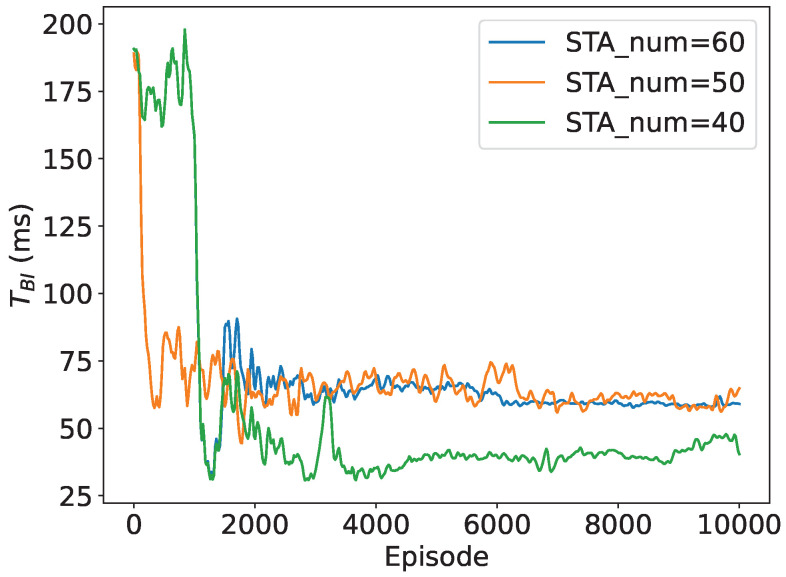
Convergence performance of $T_{BI}$ ($w.r.t.\;T_{slot}$).

Additionally, we depict the convergence performance of the slot count within a RAW and the slot duration with different numbers of STAs in the network. To provide a more straightforward demonstration, we calculate the approximate duration of a beacon interval as $T_{BI}=N_{RAW}\cdot K\cdot T_{slot}$, and we use beacon interval dynamics to represent variations in slot duration in the following subsections. As shown in Figure 7 and Figure 8, both parameters converge to stable values for different network sizes, further validating the algorithm’s convergence. As the network size is relatively small, the number of slots is similar when the number of STAs in the network is 40, 50, and 60, respectively. The duration of the beacon interval increases by about 40% when the number of STAs in the network increases from 40 to 60, indicating that the slot duration is adaptively adjusting to the network size with DRL.

#### 5.2.2. Throughput Performance with Different Traffic Loads

In this section, we analyze the adaptive adjustment of RAW parameters obtained through the PPO method with different network loads. We assume a homogeneous traffic load of 0.05 Mbps for each STA. Therefore, the traffic load in the network increases as the number of STAs in the network increases. We observe the adaptive adjustments of RAW parameters and changes in network throughput as the number of STAs increases from 30 (10) to 90.

**Figure 9:**
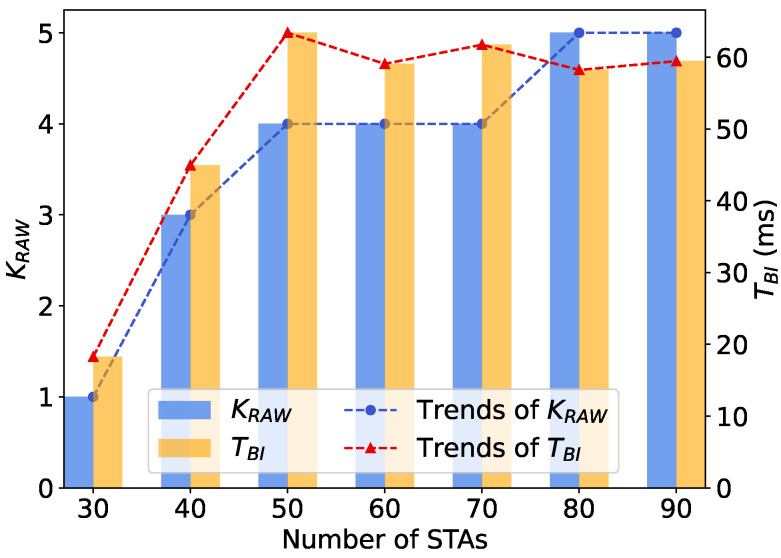
Adaptive adjustment of RAW parameters with varying network traffic loads.

As shown in Figure 9, both the slot count and slot duration increase with the growing number of STAs and the traffic load in the network. The number of slots in a RAW increases stepwise with the network size and traffic load. Specifically, the slot count remains constant when the number of STAs is between 50–70 and 80–90. This is attributed to the fact that dividing fewer slots in a RAW significantly reduces contentions when the network size is small. Overall, the RAW mechanism ensures that the number of STAs in each RAW slot is not excessive. When the number of STAs in the network is less than 50, the slot duration increases significantly from 10ms to about 50ms, approximately 4 times longer, with the increasing network size and traffic load.

**Figure 10:**
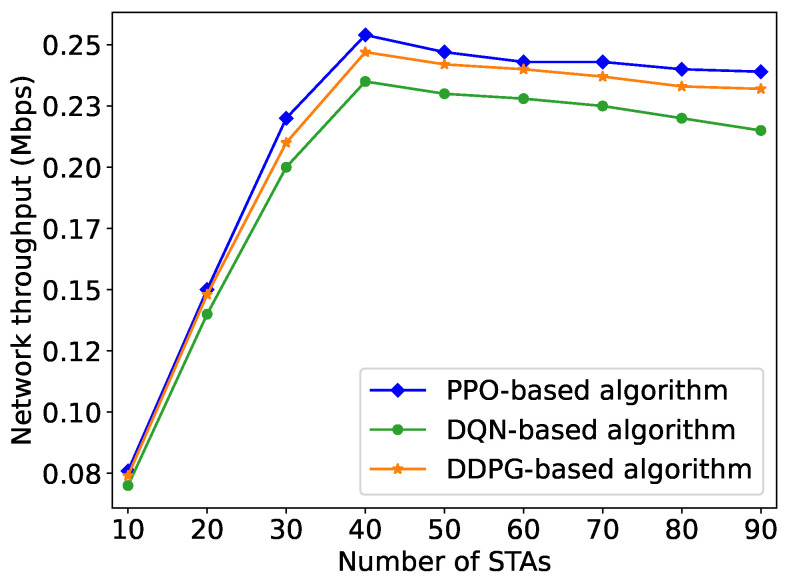
Network throughput obtained by the PPO-based algorithm with varying network traffic loads compared with the DQN-based and DDPG-based algorithms.

This trend is consistent with the changes in network throughput depicted in Figure 10. When the number of STAs in the network is less than 40, the network throughput remains unsaturated with few STAs and low traffic load in the network. As the number of STAs increases from 10 to 40, along with the ascending network traffic load, the network throughput obtained by PPO subsequently increases from 0.076Mbps to 0.248Mbps, approximately 4 times larger. At a certain point, with the number of STAs = 40, the network traffic load reaches its maximum capacity, leading to saturated network throughput under current network conditions. As the number of STAs in the network continues to increase from 40 to 90, contentions intensify, leading to a higher probability of transmission collisions. Meanwhile, when the network traffic load exceeds its capacity, constrained by the data transmission rate and the duration of a single slot, the AP cannot handle all the traffic from the STAs, resulting in a slight decrease by about 7% in network throughput. This trend is consistent with the variation of throughput with the number of STAs in IEEE 802.11ah networks [39].

We have also compared the PPO-based algorithm with DQN-based and DDPG-based algorithms. As shown in Figure 10, when the number of STAs exceeds 40, the network throughput becomes saturated. Given 90 STAs, PPO obtains 11.2% and 3.1% higher network throughput compared to DQN and DDPG, respectively. Additionally, PPO and DDPG outperform DQN in small-size networks with periodic traffic. This is because DQN is designed for discrete actions, while PPO and DDPG are for continuous actions that perform better in high action spaces.

### 5.3. Learning Performance in Random Traffic Networks

To further validate the generalization ability of the proposed DRL algorithm, in this section, we modify the network conditions. While in the previous subsection, packets are transmitted at identical intervals, we now adjust the packet transmission intervals for each STA in the network.

**Figure 11:**
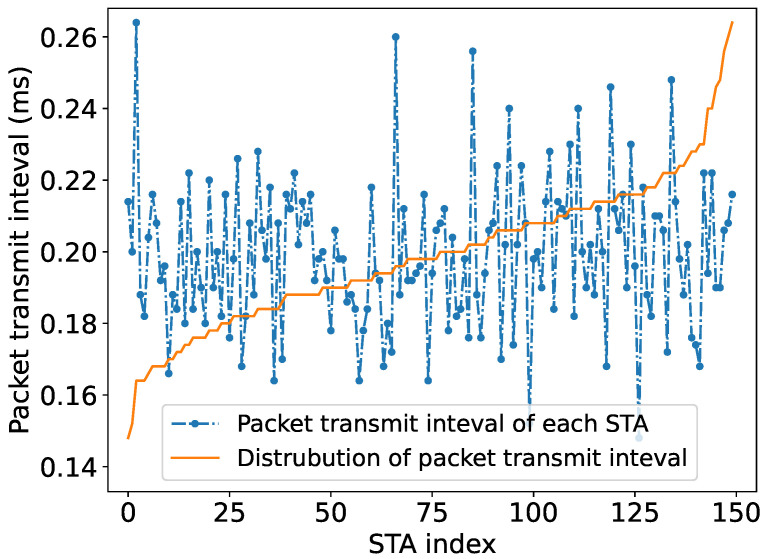
Illustration of the packet transmission interval distribution of STAs in random traffic networks.

The environment settings are shown in Table 3. In the random traffic network, the network size is set larger ($N_{max}\approx$ 300) to emulate real-world network scales. The traffic load of each STA is random, following a normal distribution with mean μ and standard deviation σ, e.g., μ=50,σ=0.1. As depicted in Figure 11, during data transmission the packet transmission interval of each STA follows a Poisson distribution with mean λ, e.g., λ=100. Additionally, we increase the maximum training iterations of the PPO agent to 20,000.

#### 5.3.1. Convergence to the Preferable RAW Parameters

We first validate the convergence performance of the PPO algorithm in the new network conditions. In the random traffic network implemented in NS-3, all the STAs transmit packets at random intervals following a Poisson distribution. Additionally, the network size is larger than that in the periodic traffic network, necessitating the division of STAs into more RAW groups. Therefore, the RAW parameters to be learned include RAW group count, slot count in one RAW, and slot duration in one RAW.

**Figure 12:**
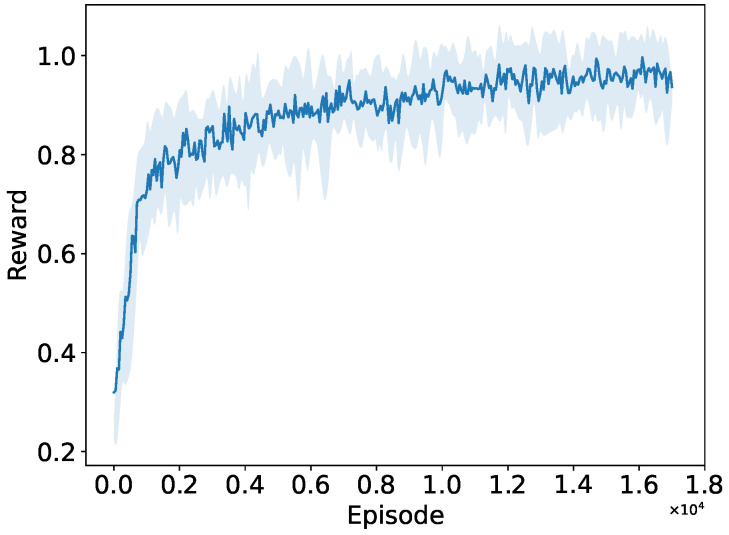
Convergence performance of the PPO algorithm in random traffic networks.

As shown in Figure 12, the normalized reward obtained by the PPO agent from interacting with the NS-3 simulation environment increases significantly as the training iterations progress, stabilizing at its maximum value after 17,000 training episodes. This indicates that the PPO agent can still learn the preferable RAW parameters and achieve optimized network throughput in the new network environment, i.e., the random traffic network. We also observe that as the network conditions become more complex, such as an increase in network size and random traffic arrivals, the PPO agent requires more interactions with the network simulation environment set up in NS-3. It needs to learn the optimal action selection strategy over twice as many training iterations in the random traffic network compared to the periodic traffic network.

**Figure 13:**
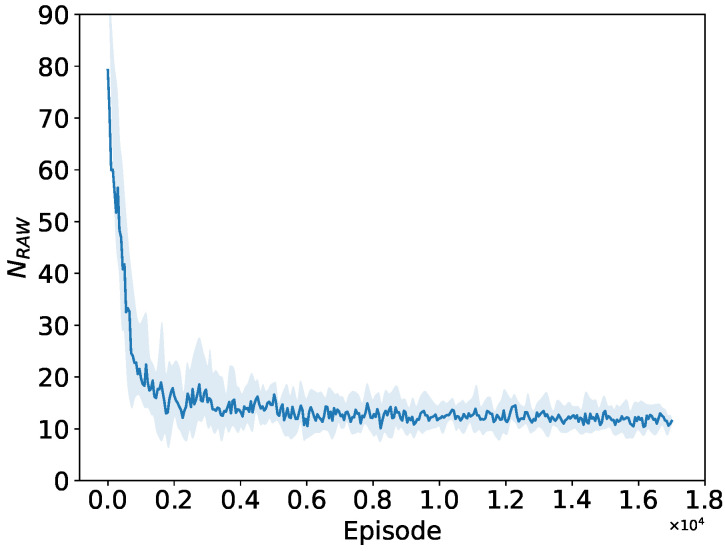
Convergence performance of RAW group count $N_{RAW}$.

**Figure 14:**
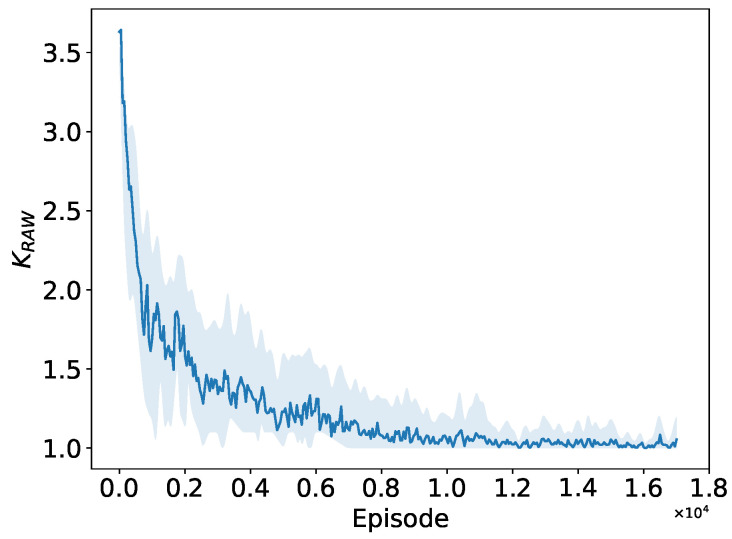
Convergence performance of slot count $K_{RAW}$.

**Figure 15:**
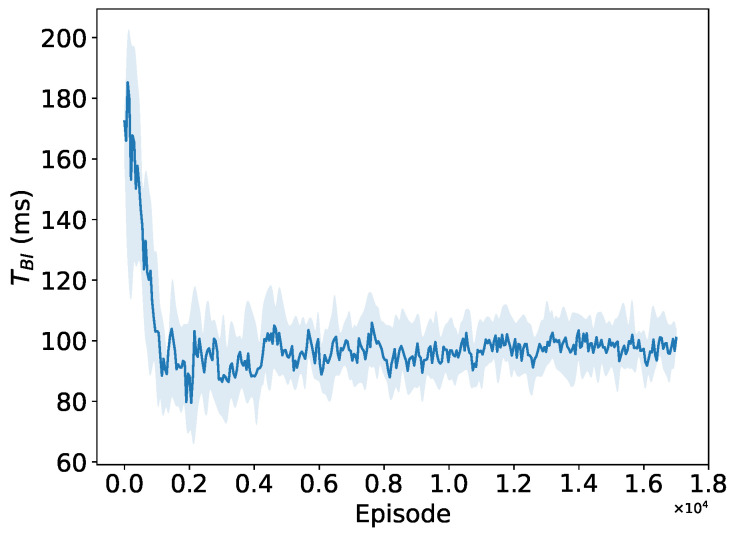
Convergence performance of BI duration $T_{BI}\;w.r.t.\;Tslot$.

As depicted in Figure 13, Figure 14 and Figure 15, when the number of STAs in the network is 150, both the number of RAW groups and the slot duration significantly increase compared to those in a small network size, while the number of slots remains small. This implies that the PPO agent tends to divide more RAW groups rather than more slots at this network size. The convergence performance to the preferable RAW parameters obtained by the PPO agent further demonstrates the generalization ability of the proposed DRL algorithm in complex networks environment with random traffic.

#### 5.3.2. Throughput Performance with Different Network Sizes

**Figure 16:**
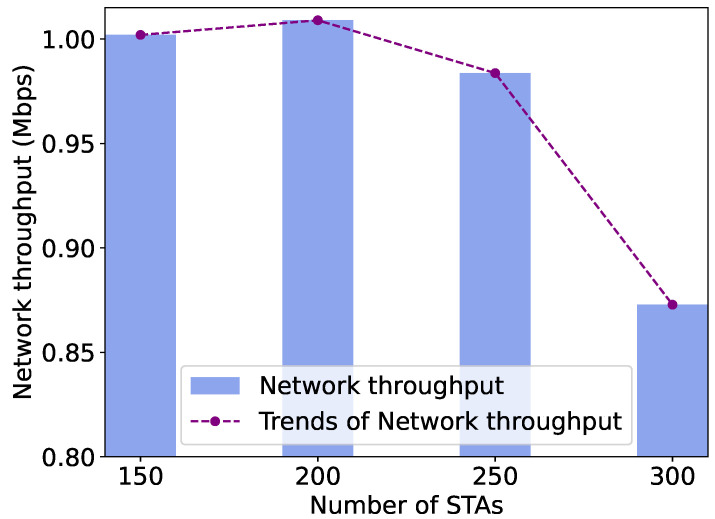
Network throughput obtained by the DRL algorithm with varying network sizes.

In this subsection, we validate and analyze the adaptive adjustment of RAW parameters and the network throughput obtained using the PPO algorithm under different network sizes, which is reflected by changes in the number of STAs. We increase the number of STAs from 150 to 300, a sufficiently large number to achieve saturated or oversaturated traffic load, which is suitable for validating the adjustment capability of RAW parameters and the network throughput of the proposed DRL framework. We observe the adaptive changes in the RAW parameters learned by the PPO agent and the network throughput obtained from the NS-3 simulation environment as the number of STAs increases. As shown in Figure 16, given that traffic load reaches saturation in large-scale networks, with the number of STAs in the network increasing from 150 to 300, the network throughput decreases by about 13%. It is evident that the increasing network size leads to intensified contention and collisions, thereby resulting in a significant decline in network throughput.

**Figure 17:**
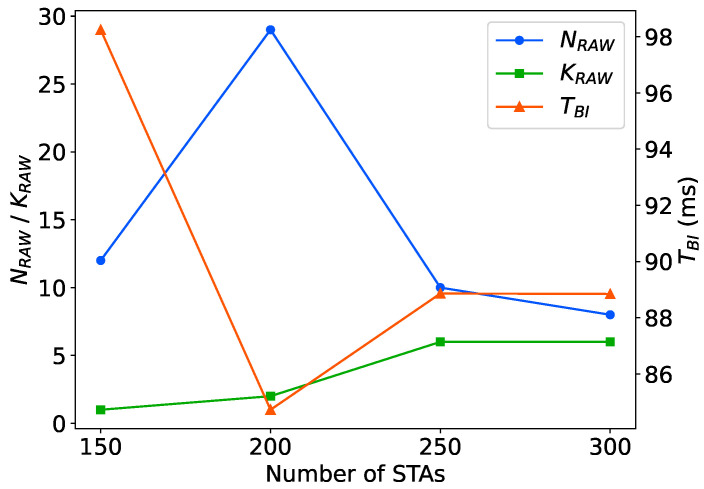
Adaptive adjustment of RAW parameters with varying network sizes.

We also provide figures to show how the RAW parameters are adjusted to maintain certain network throughput in different network sizes with over-saturated traffic loads, emphasizing the importance of adjusting preferable RAW parameters to enhance network throughput with varying network sizes. As shown in Figure 17, we observe that when the number of STAs ranges from 150 to 200, the PPO agent tends to divide STAs into roughly 3 times more RAW groups. However, when the number of STAs increases to 250–300, the agent leans towards dividing more slots (from 1 to 5) in each RAW group. We analyze that within a certain range of network sizes, simply dividing RAW groups is capable of handling the current traffic load and mitigating contention. However, as the network size grows, it becomes necessary to both divide RAW groups and more slots within each RAW group. The adaptive adjustment strategy learned by the PPO agent reduces the contention among STAs per slot, thus ensuring network throughput. Additionally, as the network size increases, the agent prefers to shorten the slot duration, consequently reducing the beacon interval duration by about 10%. We analyze that shortening the beacons broadcasting period allows the AP to schedule STAs more frequently for uplink data transmissions, thereby maintaining network throughput in intensified network conditions. We also notice that as the number of STAs increases from 60 to 150, the BI duration obtained by DRL increases from 60ms to 100ms, approximately by 66%, and the slot count increases significantly by about 3 times compared to the network size in the periodic traffic network. It is evident that as the network size scales up, contentions between STAs in the network intensify. To alleviate collisions and ensure network throughput, the PPO agent tends to dividing more slots, leading to an overall increase in the BI duration.

### 5.4. Throughput Comparison of Different Slot Division Schemes

To further demonstrate the improvement in network performance achieved by the PPO-based algorithm, we compare the network throughput obtained from the PPO-based slot division scheme with two baseline slot division schemes. In the no-slot division scheme, all STAs contend for channel access in the same RAW group without slot division. Conversely, in the equal-slot division scheme, each STA is allocated one slot in every RAW group, ensuring non-contention-based access where only one STA can access the channel in a slot.

**Figure 18:**
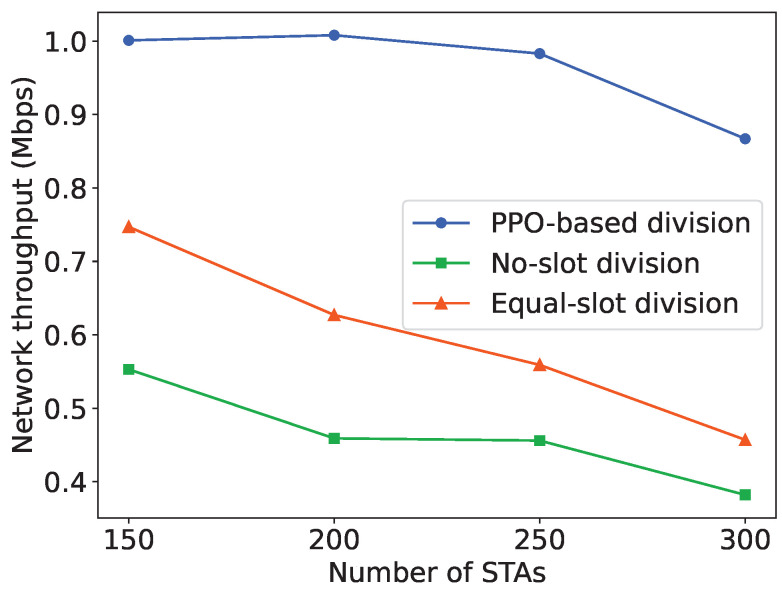
Comparison of network throughput between the PPO-based slot division scheme and the baseline slot division schemes.

The overall BI durations are the same among different slot division schemes, as determined by the BI duration learned by the PPO agent. In this case, the slot durations vary among different schemes due to different slot division methods. As depicted in Figure 18, the throughput performance obtained from the NS-3 simulation environment with the RAW slot division scheme learned by the PPO agent significantly outperforms the two basic schemes, as the number of RAW slots and slot duration are adaptively adjusted according to the network size. In the worst case, where the number of STAs in the network is 300, the network throughput obtained from the PPO-based slot division scheme is still improved by about 80% and 1.3 times, respectively, compared to the two slot division schemes. This further illustrates the effective adjustments made by PPO in Figure 17, emphasizing that the optimization of RAW parameters can significantly improve network throughput, highlighting the necessity of RAW parameters optimization.

It can be observed that as the network size increases, contentions and collisions among STAs in the network intensify, leading to a decrease in network throughput for all three slot division schemes. However, when the number of STAs in the network is between 150 and 250, the decrease in network throughput obtained from the PPO-based slot division scheme in the NS3 simulation environment is much smaller than that of the other two schemes. This indicates that the learning-based RAW slot division scheme can maintain better network throughput than the basic slot division schemes in deteriorating network conditions. It can be validated that the PPO agent can learn the preferable RAW parameters and effectively improve the network throughput, especially in scenarios with high contention. We also observe that as the network size increases, the network performance obtained by the division scheme that allocates one slot to each STA is significantly better than that of the scheme that does not divide slots within a RAW. This further validates the necessity of using the RAW grouping mechanism in large-scale networks and its improvement on network performance.

## 6. Conclusions

In this paper, we have proposed a PPO-based DRL algorithm for optimizing RAW parameters in the IEEE 802.11ah-based IoT network. Necessary analysis was first provided to emphasize the significant impact of RAW parameters on network throughput, and the RAW parameters optimization problem was formulated. A DRL framework interacting with the NS-3 simulator was then proposed, in which the optimization problem was reformulated as an MDP, and a PPO-based algorithm for RAW parameters optimization was proposed. In network environments with periodic and random traffic built in the NS-3 simulator, the performance of the proposed DRL algorithm was evaluated. The simulation results show that the PPO-based DRL scheme can adaptively adjust RAW parameters under different network conditions and achieve significantly improved network throughput compared to that of the baseline slot division schemes.

The proposed DRL and NS-3 simulation framework can be extended to different IEEE 802.11ah IoT network scenarios and optimization problems, such as the design of channel access mechanisms. Channel access optimization is particularly important in complex scenarios involving diverse traffic types, expanding network scales, and dynamic network topologies. In addition, complex network conditions hinder rapid environment reconstruction in NS-3, reducing learning and interaction efficiency in DRL. To reduce interaction overhead and enable real-time application, it is beneficial to develop a more accurate and comprehensive channel access analytical model. However, analytical models constructed solely relying on mathematical methods is limited when solving problems involving the joint optimization of multiple mechanisms. To address this limitation, a lightweight “surrogate” model can be constructed by collecting test data from real deployed IEEE 802.11ah IoT scenarios and fitting them using statistical methods and AI techniques. This model would be adaptable to diverse scenarios and capable of efficiently interacting with DRL algorithms. Moreover, in networks with time-varying network sizes and heterogeneous traffic, RAW grouping problems involve categorizing STAs into different RAW groups, requiring optimizing grouping strategies. In this paper, we have demonstrated the ability of the DRL approach to effectively determine the preferable RAW parameters across different network environments. Consequently, the DRL approach can be extended to address the challenge of finding the optimal RAW grouping strategy.

## Figures and Tables

**Table 1:** Parameter settings in periodic traffic networks.

Parameters	Settings
Wi-Fi channel configuration	MCS 0, 2 MHz
coverage radius	300 m
data rate	650 kbit/s
traffic type	UDP
payload size	100 bytes
network size *N*	small, 60 (basic setting)
number of RAW group	1 (basic setting)
traffic load of the STAs	same
set of traffic loads D	$d_{1}=\ldots =d_{N}$
packet transmit interval of the STAs	periodic (same)
set of traffic intervals I	$i_{1}=\ldots =i_{N}$

**Table 2:** Parameter settings for DRL training.

Parameters	Settings
state dimension	1
action dimension	100 × 63 × 2047
discount factor	0.99
generalized advantage estimation factor	0.95
PPO clip rate	0.2
PPO update times/epochs	10
max training episodes	1 × $10^{4}$ (default)
hidden net width	256
learning rate of actor	3 × $10^{-4}$
learning rate of critic	3 × $10^{-4}$
L2 regularization coefficient for critic	1 × $10^{-3}$
types of probability distribution of actor	beta
length of sliced trajectory of actor	64
length of sliced trajectory of critic	64
entropy coefficient of actor	0
decay rate of entropy coefficient of actor	0.9998

**Table 3:** Parameter settings in random traffic network.

Parameters	Settings
Wi-Fi channel configuration	MCS 0, 2 MHz
coverage radius	300 m
data rate	650 Kbit/s
traffic type	UDP
payload size	100 bytes
network size *N*	large, 150 (basic setting)
traffic load of the STAs	random
set of traffic loads D	$d_{n}∼N\left(\mu ,\sigma^{2}\right),\;n\in \left[1,N\right]$
packet transmission interval of the STAs	random
set of traffic intervals I	$i_{n}∼\pi \left(\lambda \right),\;n\in \left[1,N\right]$

## Data Availability

Data are contained within the article.
